# Drunken Women

**Published:** 1868-08

**Authors:** 


### DRUNKEN WOMEN,

Or "Intemperate Ladies," as a cotemporary more elegantly expresses it; but it is better to speak of evils so as to bring them up before the mind in all their startling deformity; still we recoil at the heading, and are conscious of an inner effort to persuade ourselves that there is not such a thing in existence as a "drunken woman." My mother drunk? my sister? my daughter? my wife? Impossible! yes, impossible to me, but not to all.

One of the handsomest young wives in New-York, and of a family name, which, as to social position, was second to none, died of an over-draught of her favorite stimulus. In default of other supplies, she frequently drank the cologne water of her toilet-table; and yet for the troubled and the sorrowing she had a heart so generous and a nature so kind, we have known her, when sudden and crushing trials came to the poor and obscure, of whom she never heard before, to visit the saddened household, speaking words of sympathy, and sharing her wardrobe with the children of sudden misfortune.

Not long ago there died the wife of a great name, whose infatuation for the cup was such, that for the last years of her life an attendant was appointed to follow her, every hour of her waking existence, and yet the heiress of hundreds of thousands died a drunkard's death!

For a summer we lived in the same house with a lady whose intellectuality shone conspicuously in any company; whose poetry, as pronounced by herself, would charm all hearers; and whose vivacity and conversational powers, were such as to compel the admiration of every stranger; and yet when the desire for drink came upon her, like a strong man armed, she hesitated not to practice the most degrading deceptions, to feign the most fearful diseases, that in the absence of her accomplished husband, stimulants should be brought to her by kind and unsuspecting attendants; and then for several days she would revel in the most beastly intoxication.

An official announcement was made not long ago, that some four hundred applications had been received for admission into the asylum for inebriates, now in process of erection at Binghamton, a large portion of which were from women in the upper walks of life.

Not long ago a man of great wealth was applied to for a donation for the State Asylum; he declined on the ground that he had no personal interest in such an establishment, as there was no danger of any of his family requiring its aid. Later on, a young man of this city died in a drunken revel; it was his son, just entering manhood! The truth is, no parent can say that any son or daughter will not die an inebriate. A writer says of New-York:

"There is a great and growing evil in this city, but one of such delicate nature as to almost forbid being dragged into public print. I refer to the increasing and lamentable habit now so common, of the indulgence by ladies in intoxicating drinks. I do not refer to those who do wrong almost from necessity, but to that other class who have rich husbands and homes that might be made happy. A larger number of this class seem steadily to be diving deeper into dissipation every year, than many persons greatly interested in their welfare and happiness even imagine. I have heard recently of several distressing cases of this kind, and to-day I learn that the wife of a well-known citizen, reported to be very wealthy, has been sent to the lunatic asylum, in the hope that she may, with returning reason, be enabled to overcome the terrible temptations which intoxicating liquors have of late had for her. Her husband's name is almost as familiar in some parts of the South as it is here."

It is unwise to attribute this growing evil to any one cause, for there are a variety in operation; a few may be named, and the reader must make such use of them as an intelligent conscience may urge.

Reputable physicians seem to be falling into the habit more and more of advising alcoholic remedies, either frankly and above-board, or under the disguise of Tonics and Tinctures and Bitters. Scarcely a religious newspaper of any name or sect can be taken up, which does not contain advertisements of these same mischievous agencies with "Reverend" certifiers *ad nauseam.* The editors of respectable medical journals, and the publishers of the same lend their aid towards the introduction of wines and beers and brandies into the families by whose patronage they live; thus prostituting their influence to vile purposes for the sake of the few dollars they receive from the advertisement of the same. To show the extent to which these things are practiced, we take up a medical periodical for July, of this city, issuing from the establishment of a name, which, for half a century has commanded the respect of this whole community; the editor, an old man, of learning and culture, and high position in his profession and in his church; such men, we say, are found introducing to the knowledge of their readers "Pure Liquors for the use of the sick," and telling where such a brand of gin and such a quality of whisky can be had; showing, however, some little deference to public decency, by saying: "So long as people will take domestic medicines, they ought, at least, to discriminate the good from the bad." How is it possible to "discriminate" between good and bad London Dock Gin, and Philadelphia Whisky, and French Cordials, when all are bad; when the use of any of them for a short time *tends* to set up a desire for more, which no man of intelligence, and who has any respect for himself, or for the truth, will deny. How many men and women under the habitual use of Tonics, and Bitters, and Beers, and Cordials, have waked up at last to the fearful truth that they "can not do without them," "must have them," let our asylums, and prisons, and poor-houses testify, and let ruined families, and blasted reputations, and broken hearts from the land over, confirm the terrible record! He, and he only, is safe from a drunkard's death, who never tastes a drop of any thing that can intoxicate.

